# Cold ischemic time and early vasoactive-inotropic support following heart transplantation with HTK-preserved donor hearts

**DOI:** 10.1016/j.jhlto.2026.100669

**Published:** 2026-08-17

**Authors:** Truc Mai Nguyen, Zied Ltaief, Filip Dulguerov, Tamila Abdurashidova, Anna Nowacka, Valentina Rancati, Philippe Meyer, Léa Iten, Olivier Muller, Patrick Yerly, Roger Hullin, Matthias Kirsch, Henri Lu

**Affiliations:** a Division of Cardiology, Lausanne University Hospital, University of Lausanne, Lausanne, Switzerland; b Department of Adult Intensive Care Medicine, Lausanne University Hospital, University of Lausanne, Lausanne, Switzerland; c Division of Cardiac Surgery, Lausanne University Hospital, University of Lausanne, Lausanne, Switzerland; d Division of Anesthesiology, Lausanne University Hospital, University of Lausanne, Lausanne, Switzerland; e Division of Cardiology, Geneva University Hospital, University of Geneva, Geneva, Switzerland; f Division of Cardiovascular Medicine, Brigham and Women’s Hospital, Harvard Medical School, Boston, MA

**Keywords:** heart transplantation, cold ischemic time, vasoactive-inotropic score, graft preservation, ischemia-reperfusion injury

## Abstract

Whether longer cold ischemic time (CIT) increases early hemodynamic support after heart transplantation (HTx) with histidine-tryptophan-ketoglutarate (HTK) preservation is unclear. We examined the association between CIT and the vasoactive-inotropic score (VIS), a marker of circulatory support. We studied consecutive adults transplanted with HTK-preserved grafts at Lausanne University Hospital (January 2016–March 2025). VIS was calculated hourly for 48 h after ICU admission, and its association with CIT modeled using restricted cubic splines. Among 130 recipients (75% male, aged 52±12 years), mean CIT was 152±50 min. Longer CIT was associated with higher 24-hour VIS (P_overall_=0.048), rising steeply beyond ≈200 min. Patients with CIT ≥200 min (n=14) had higher VIS (difference at 48 h 10.2 points, p=0.024), more post-transplant ECMO (43% vs 9%, p<0.001), and longer ICU stay (11.0 vs 6.1 days, p=0.014). The CIT–VIS relationship was non-linear, supporting CIT as a potentially modifiable determinant of early hemodynamic status after HTx.

## Graphical abstract

**Figure:**
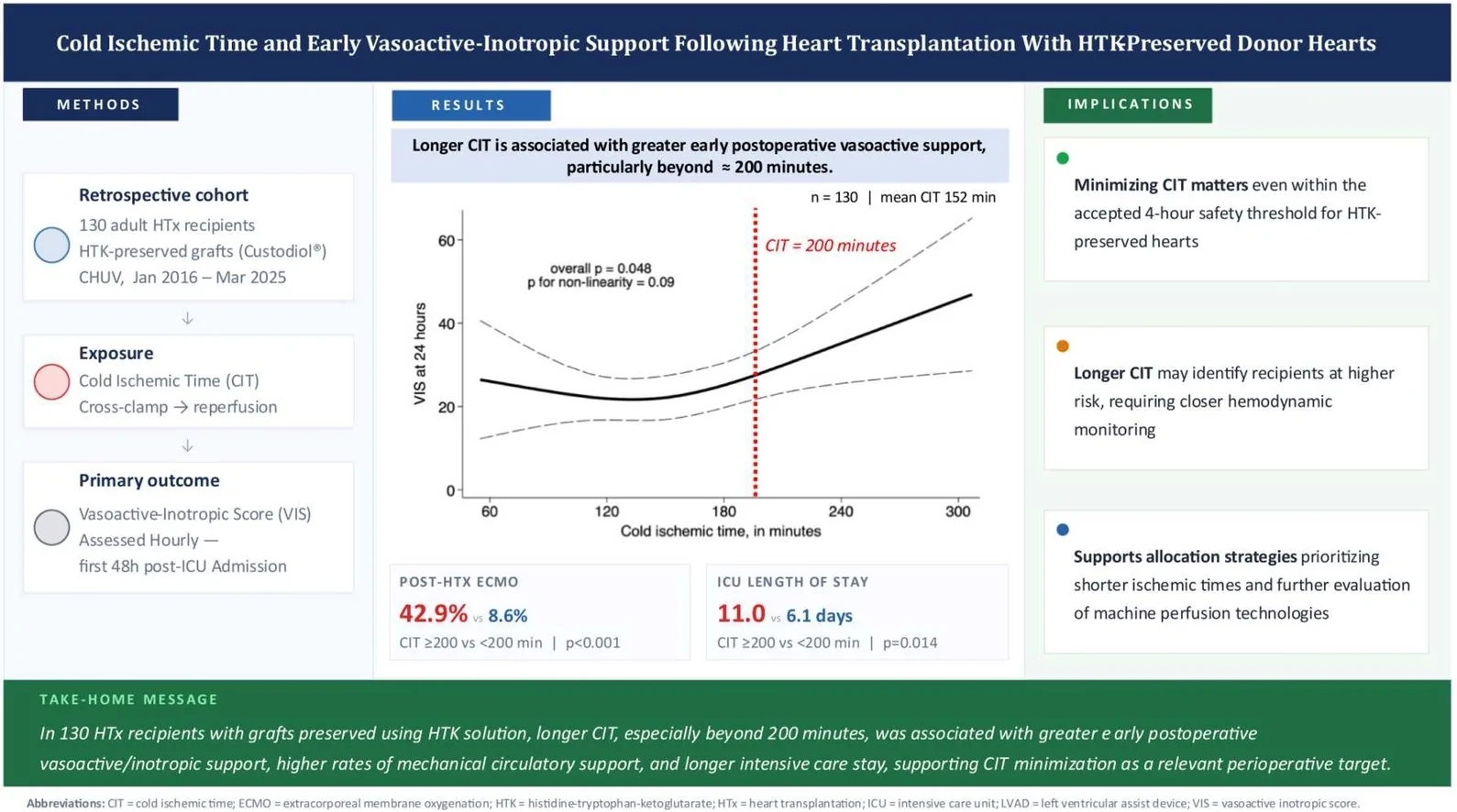

## Introduction

Prolonged cold ischemic time (CIT) is an established risk factor for graft dysfunction and mortality after heart transplantation (HTx). In studies of hearts preserved with histidine–tryptophan–ketoglutarate (HTK) solution, outcomes have generally been acceptable within 4 h of ischemia, whereas longer preservation has been associated with poorer outcomes.^1^, ^2^, ^3^ The vasoactive-inotropic score (VIS), which provides a graded, continuously quantifiable measure of pharmacological circulatory support, is also associated with higher morbidity and mortality after HTx.^4^

Less well characterized is the continuous relationship between CIT and early vasoactive-inotropic requirements. We therefore examined the association between CIT and serial VIS during the first 48 h after HTx in a single-center cohort in which all donor hearts were preserved with HTK.

## Methods

We conducted a retrospective single-center study including consecutive adults who underwent HTx with an HTK-preserved donor heart at Lausanne University Hospital between January 2016 and March 2025. All donor hearts were procured from brain-dead donors and transported using static cold storage. Patients aged <18 years, recipients of grafts preserved using normothermic machine perfusion, and those with unavailable CIT were excluded. The study was approved by the local ethics committee (CER-VD 2019–00704).

CIT was defined as the interval between donor aortic cross-clamping and graft reperfusion. Recipient characteristics reflected the latest available measurements before transplantation.

The VIS was calculated hourly during the first 48 h after intensive care unit (ICU) admission as previously described.^5^ Patients who died within the 48-hour window were excluded from the serial VIS analyses but retained in all other analyses; VIS values were not imputed after death. The primary outcome was VIS at 6, 12, 24, and 48 h. Secondary outcomes included post-transplant extracorporeal membrane oxygenation (ECMO), duration of mechanical ventilation, ICU and hospital length of stay, renal function, and survival.

CIT was modeled continuously using restricted cubic splines with three knots, adjusted for recipient age, donor age, female-donor-to-male-recipient mismatch, and pretransplant left ventricular assist device (LVAD). A post-hoc spline-derived threshold dichotomized the cohort for exploratory comparisons. Survival was described using Kaplan–Meier estimates and univariable Cox regression. A two-sided P value <0.05 was considered statistically significant. Analyses used Stata 19 (StataCorp, College Station, TX).

## Results

Of 141 eligible patients, 6 were excluded for normothermic machine perfusion and 5 for insufficient CIT documentation, leaving 130. The cohort was 75% male (mean age 52±12 years), 42% had a pretransplant LVAD, mean CIT was 152±50 min (Table 1). One patient died within 48 h and was excluded from the VIS analyses.

**Table 1:** Patients’ Baseline (Pre-transplant) Characteristics

	**n=130**
Sex (male)	98 (75.4)
Age, years	52.5±11.7
Weight, kg	78.1±14.0
Height, m	1.7±0.1
BMI, kg/m^2^	26.4±4.3
Ischemic HF Etiology	68 (52.3)
NT-proBNP, ng/mL	1539 [686, 3177]
Diabetes	37 (28.5)
Hypertension	50 (38.5)
Cirrhosis	2 (1.5)
CKD	30 (23.1)
Creatinine, µmol/L	119±87
eGFR, mL/min/1.73 m^2^	28.9±13.5
Dialysis	4 (3.1)
COPD	7 (5.4)
Sleep apnea	30 (23.1)
LVEF, %	26±12
Pre-transplant LVAD	55 (42.3)
CRT	48 (36.9)
ICD	38 (29.2)
CIT, min	152±50
*Hemodynamic data*	
Peak VO_2_, mL/kg/min	13.6±4.9
mPAP, mmHg	28.0±10.7
mPVR, WU	2.5±1.9

Results are expressed as n (%), mean ± standard deviation, or median [25th percentile, 75th percentile].

All results refer to the transplant recipient.

Abbreviations: BMI = body mass index; CIT = cold ischemic time; CKD = chronic kidney disease; COPD = chronic obstructive pulmonary disease; CRT = cardiac resynchronization therapy; eGFR = estimated glomerular filtration rate; ICD = implantable cardioverter-defibrillator; LVAD = left ventricular assist device; LVEF = left ventricular ejection fraction; mPAP = mean pulmonary artery pressure; mPVR = mean pulmonary vascular resistance; NT-proBNP = N-terminal pro–B-type natriuretic peptide; VO₂ = oxygen consumption; WU = Wood units.

**Figure 1:**
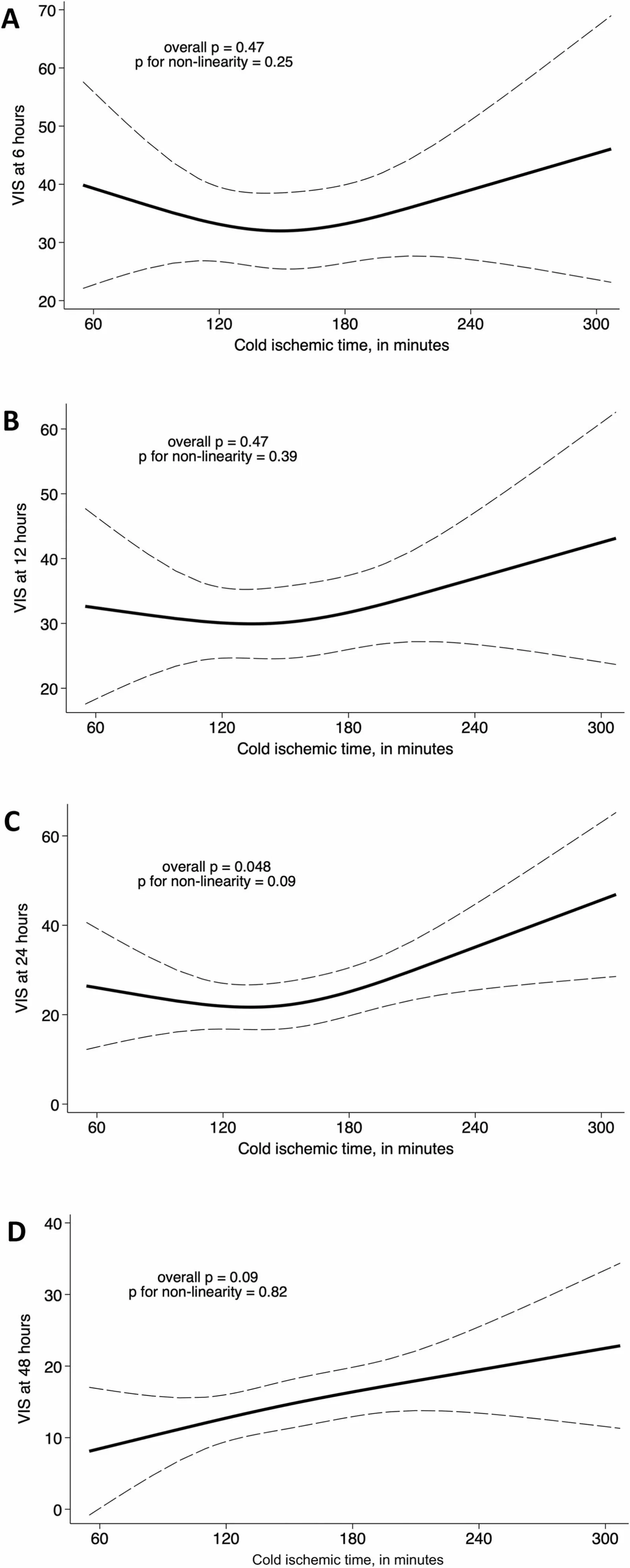
Association between cold ischemic time and vasoactive-inotropic score at (A) 6 h, (B) 12 h, (C) 24 h, and (D) 48 h after ICU admission (n=129). All analyses were adjusted for recipient age, donor age, female-donor-to-male-recipient mismatch, pre-transplant LVAD. Abbreviations: ICU = intensive care unit; LVAD = left ventricular assist device; VIS = vasoactive-inotropic score.

At 6 and 12 h, VIS remained stable up to approximately 150 min of CIT before rising with longer ischemia, without reaching significance (P_overall_=0.47 for both; Figure 1A–B). At 24 h the association was significant (P_overall_=0.048), with VIS increasing progressively beyond 150 min and more steeply beyond approximately 200 min (Figure 1C); the pattern of the spline was consistent using four knots (Figure S1). At 48 h the relationship appeared predominantly linear and did not reach significance (P_overall_=0.09; Figure 1D).

**Figure 2:**
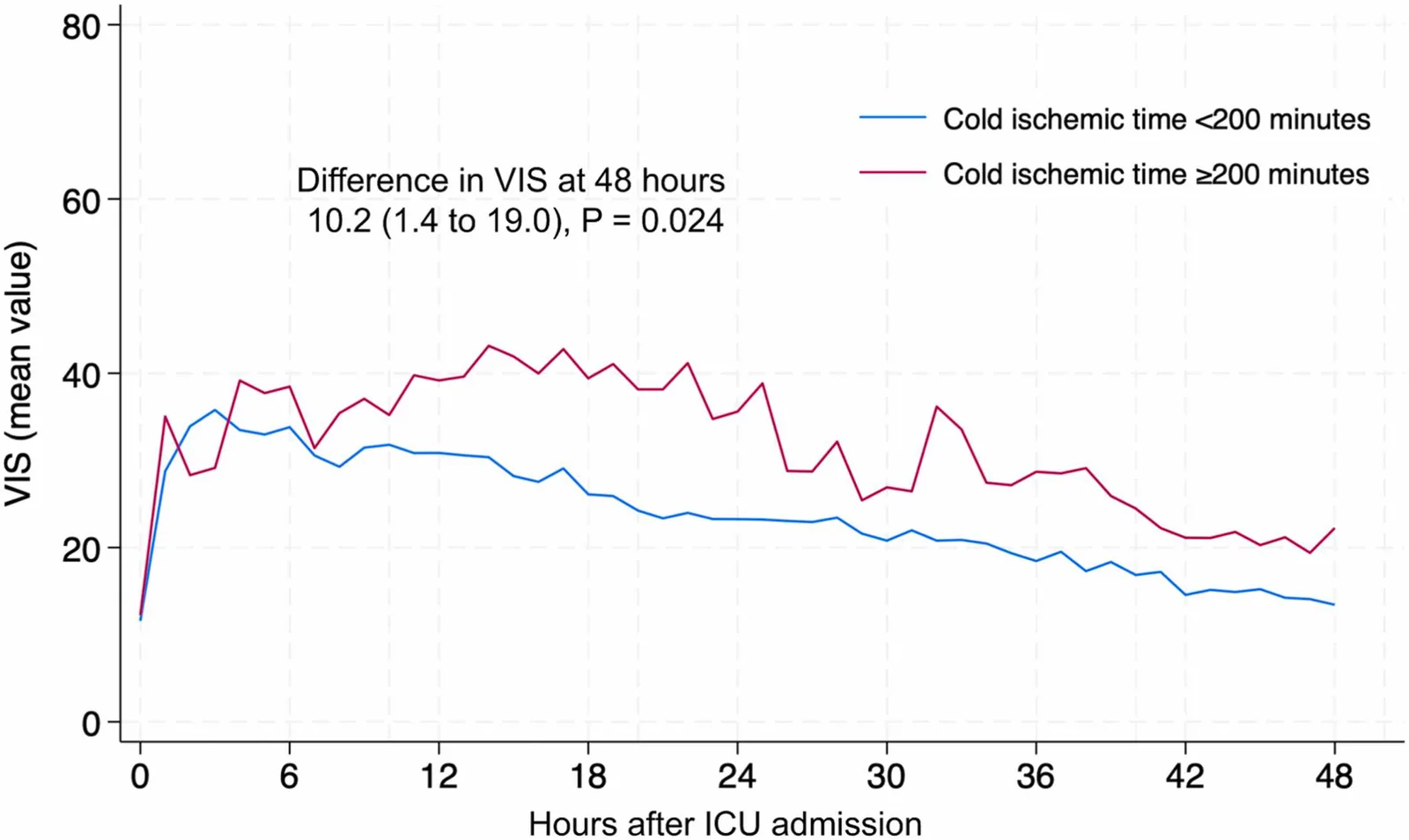
Vasoactive-inotropic score trajectory during the first 48 h after ICU admission according to cold ischemic time category (<200 vs. ≥200 min) (n=129). Difference in VIS at 48 h after ICU admission estimated using linear regression, adjusted for recipient age, donor age, female-donor-to-male-recipient mismatch, and pre-transplant LVAD support. Abbreviations: ICU = intensive care unit; LVAD = left ventricular assist device; VIS = vasoactive-inotropic score.

The 24-hour spline suggested an inflection near 200 min. Patients were therefore grouped post hoc into two categories: CIT ≥200 min (n=14, 11%) versus <200 min (Table S1). Over a median follow-up of 2.0 years, all-cause mortality occurred in 3 patients (21%) with CIT ≥200 minutes ≥200 min and 13 (11%) with CIT <200 minutes <200 min (HR 1.90, 95% CI 0.54 6.70; Table S2, Figure S2). The ≥200-minute group had higher VIS across most of the 48-hour period, with an adjusted difference at 48 h of 10.2 points (95% CI 1.4–19.0; p=0.024; Figure 2). They also more frequently required post-transplant ECMO (43% vs 9%, p<0.001), had longer mechanical ventilation (5.0 [2.3–14.3] vs 2.1 [0.7–5.0] days, p=0.005) and longer ICU stay (11.0 [6.0–20.9] vs 6.1 [3.9–10.5] days, p=0.014); hospital stay did not differ (p=0.39). Sequential Organ Failure Assessment scores were higher at ICU admission (13.0±3.1 vs 10.3±3.1, p=0.023) and at 48 h (p=0.033), while renal function was similar (Table S3).

**Figure 3:**
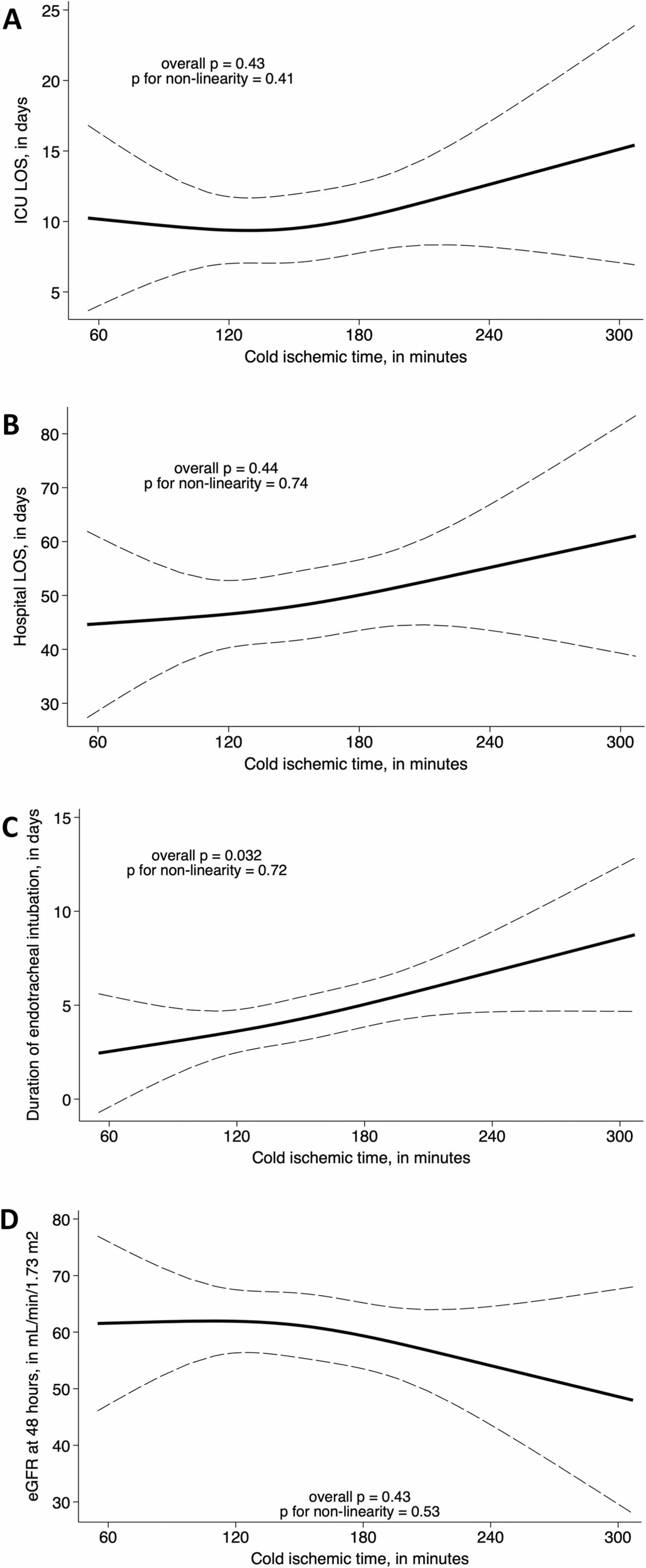
Association between cold ischemic time and (A) ICU LOS, (B) hospital LOS, (C) duration of endotracheal intubation, and (D) eGFR 48 h after ICU admission (n=130). All analyses were adjusted for recipient age, donor age, female-donor-to-male-recipient mismatch, pre-transplant LVAD. Association between cold ischemic time and eGFR is further adjusted for eGFR at baseline (pre-transplant) and dialysis after transplant. Abbreviations: eGFR = estimated glomerular filtration rate; ICU = intensive care unit; LOS = length of stay; LVAD = left ventricular assist device.

Longer CIT was associated with prolonged ventilation (P_overall_=0.032) but not with ICU or hospital stay, or with 48-hour estimated glomerular filtration rate (Figure 3).

## Discussion

In this cohort of 130 HTK-preserved recipients, longer CIT was associated with greater early vasoactive-inotropic requirements in a non-linear fashion, with VIS rising from approximately 150 min and more sharply beyond 200—well below the accepted 4-hour limit. Recipients above this exploratory threshold also more often required post-transplant ECMO and experienced longer mechanical ventilation and ICU stays, pointing to a broader pattern of early postoperative severity rather than an isolated increase in vasoactive support.

Although CIT and VIS are both established prognostic markers after HTx, our study characterizes the continuous relationship between them and identifies a signal within the conventional 4-hour window. These findings align with Venema et al., where the association between early inotrope support and outcome was attenuated after adjustment for ischemia time, suggesting that ischemia contributes to inotrope burden.^6^ They also accord with registry data placing the risk inflection before 4 h: in more than 36,000 UNOS transplants, Tang et al. identified a threshold near 3 h, with rising risk thereafter.^7^

These results should also be considered in the context of prior HTK studies. HTK generally provides effective myocardial protection within 4 h but has appeared less favorable than University of Wisconsin solution as ischemic time approaches this limit,^8^ whereas meta-analytic data suggest only modest survival differences among preservation solutions.^9^ By restricting the cohort to HTK-preserved grafts, we reduced between-solution heterogeneity, although the findings may not extend to other preservation strategies. The steeper VIS increase beyond 200 min is compatible with progressive ischemia-reperfusion injury during cold storage, supporting further evaluation of machine perfusion.^10^

This single-center retrospective study has limited generalizability. The 200-minute threshold was exploratory rather than validated, and the few patients above it limited power for secondary and mortality analyses. Residual confounding from unmeasured procurement or intraoperative factors cannot be excluded, and findings are specific to HTK preservation.

## Conclusion

Among 130 HTx recipients with HTK-preserved grafts, longer CIT was associated with greater early vasoactive-inotropic support below the conventional 4-hour limit. Whether minimizing ischemia within this window improves early hemodynamic recovery warrants prospective evaluation.

## Funding sources

None.

## Financial Disclosure Statement

None.

## Declaration of Competing Interest

The authors declare that they have no known competing financial interests or personal relationships that could have appeared to influence the work reported in this paper.

## Footnotes

Appendix A Supplemental data associated with this article can be found in the online version at doi:10.1016/j.jhlto.2026.100669.
