# Supplementary material for: Cold ischemic time and early vasoactive-inotropic support following heart transplantation with HTK-preserved donor hearts
Source: JHLT Open. 2026 Aug 17;14:100669. doi: 10.1016/j.jhlto.2026.100669 (PMC13544163; doi:10.1016/j.jhlto.2026.100669)
Supplement: Supplementary file 1 [file mmc1.docx]

**Supplemental data**

**Cold Ischemic Time and Early Vasoactive-Inotropic Support Following Heart Transplantation With HTK-Preserved Donor Hearts**

Truc Mai Nguyen, Zied Ltaief, Filip Dulguerov, Tamila Abdurashidova, Anna Nowacka, Valentina Rancati, Philippe Meyer, Léa Iten, Olivier Muller, Patrick Yerly, Roger Hullin, Matthias Kirsch, Henri Lu

**Table S1.** Patients’ baseline (pre-transplant) characteristics according to cold ischemic time (≥200 vs. <200 minutes)

**Table S2.** Event numbers, event rates, and hazard ratio associated with cold ischemic time (≥200 vs. <200 minutes)

**Table S3**. Patients’ postoperative outcomes according to cold ischemic time (≥200 vs. <200 minutes)

**Figure S1**. Adjusted association between cold ischemic time and vasoactive-inotropic score at 24 hours after ICU admission using a 4-knot spline model (n=129)

**Figure S2**. Overall survival by cold ischemic time (≥200 vs. <200 minutes)

**Table S1.** Patients’ baseline (pre-transplant) characteristics according to cold ischemic time (≥200 vs. <200 minutes)

|  | **CIT <200 min (n=116)** | **CIT ≥200 min (n=14)** | **P-value** |
| --- | --- | --- | --- |
| Sex (male) | 89 (76.7) | 9 (64.3) | 0.31 |
| Age, years | 52.3 ± 11.7 | 53.7 ± 12.2 | 0.68 |
| Weight, kg | 78.6 ± 13.6 | 73.8 ± 17.1 | 0.22 |
| Height, m | 1.7 ± 0.1 | 1.7 ± 0.1 | 0.20 |
| BMI, kg/m^2^ | 26.4 ± 4.1 | 25.7 ± 5.8 | 0.56 |
| Ischemic HF Etiology | 61 (52.6) | 7 (50.0) | 0.85 |
| NT-proBNP, ng/mL | 1,559 [686, 3,177] | 1,325 [711, 3,158] | 0.82 |
| Diabetes | 34 (29.3) | 3 (21.4) | 0.54 |
| Hypertension | 45 (38.8) | 5 (35.7) | 0.82 |
| Cirrhosis | 2 (1.7 ) | 0 (0.0 ) | 0.62 |
| CKD | 26 (22.4) | 4 (28.6) | 0.61 |
| Creatinine, µmol/L | 120 ± 91 | 107 ± 34 | 0.61 |
| eGFR, mL/min/1.73 m^2^ | 29 ± 14 | 29 ± 14 | 0.96 |
| Dialysis | 4 (3.4) | 0 (0.0) | 0.48 |
| COPD | 6 (5.2) | 1 (7.1) | 0.76 |
| Sleep apnea | 26 (22.4) | 4 (28.6) | 0.61 |
| LVEF, % | 27 ± 12 | 26 ± 10 | 0.77 |
| Pre-transplant LVAD | 51 (44.0) | 4 (28.6) | 0.27 |
| CRT | 42 (36.2) | 6 (42.9) | 0.63 |
| ICD | 36 (31.0) | 2 (14.3) | 0.19 |
| CIT, min | 140 ± 35 | 255 ± 42 | By design |
| *Hemodynamic data* |  |  |  |
| Peak VO_2_, mL/kg/min | 13.0 ± 4.2 | 18.0 ± 7.4 | 0.001 |
| mPAP, mmHg | 28.0 ± 10.5 | 27.5 ± 12.3 | 0.88 |
| mPVR, WU | 2.5 ± 1.7 | 3.2 ± 3.2 | 0.2 |

Results are expressed as n (%), mean ± standard deviation, or median [25^th^ percentile, 75^th^ percentile].

All results refer to the transplant recipient.

Abbreviations: BMI = body mass index; CIT = cold ischemic time; CKD = chronic kidney disease; COPD = chronic obstructive pulmonary disease; CRT = cardiac resynchronization therapy; eGFR = estimated glomerular filtration rate; ICD = implantable cardioverter-defibrillator; LVAD = left ventricular assist device; LVEF = left ventricular ejection fraction; mPAP = mean pulmonary artery pressure; mPVR = mean pulmonary vascular resistance; NT-proBNP = N-terminal pro–B-type natriuretic peptide; VO₂ = oxygen consumption; WU = Wood units.

**Table S2**. Event numbers, event rates, and hazard ratio associated with cold ischemic time (≥200 vs. <200 minutes)

|  | **Events, n (%)** | **Event rate**  **(95% CI), per 100 py** | **Unadjusted HR**  **(95% CI)** |
| --- | --- | --- | --- |
| **All-cause death** | | | |
| - CIT <200 min (n=116) | 13 (11.2) | 3.8 (2.2-6.6) | 1 (reference) |
| - CIT ≥200 min (n=14) | 3 (21.4) | 7.6 (2.5-23.7) | 1.90 (0.54-6.70) |

Median follow-up: 2.0 (IQR 1.3-5.4) years

Abbreviation: CIT = cold ischemic time.

**Table S3.** Patients’ postoperative outcomes according to cold ischemic time (≥200 vs. <200 minutes).

|  | **CIT <200 min (n=116)** | **CIT ≥200 min (n=14)** | **P-value** |
| --- | --- | --- | --- |
| Duration of mechanical ventilation, in days | 2.1 [0.7, 5.0] | 5.0 [2.3, 14.3] | 0.005 |
| ICU LOS, in days | 6.1 [3.9, 10.5] | 11.0 [6.0, 20.9] | 0.014 |
| Hospital LOS, in days | 37.7 [29.9, 52.3] | 41.8 [33.0, 77.7] | 0.39 |
| Post-transplant ECMO | 10 (8.6) | 6 (42.9) | <0.001 |
| Post-transplant ECMO duration, in days | 6.9 [2.5, 8.8] | 4.5 [2.6, 6.5] | 0.81 |
| eGFR at hour 24, in mL/min/1.73 m^2^ | 59.4 ± 29.1 | 51.8 ± 20.1 | 0.35 |
| eGFR at hour 48, in mL/min/1.73 m^2^ | 60.4 ± 33.8 | 47.9 ± 20.8 | 0.18 |
| SOFA score at hour 0 | 10.3 ± 3.1 | 13.0 ± 3.1 | 0.023 |
| SOFA score at hour 24 | 10.5 ± 3.0 | 12.9 ± 4.9 | 0.05 |
| SOFA score at hour 48 | 10.3 ± 3.8 | 13.5 ± 5.6 | 0.033 |

Results are expressed as mean ± standard deviation, or median [25^th^ percentile, 75^th^ percentile].

SOFA scores were missing in 49 patients (37.7%). ECMO duration was calculated among patients requiring post-transplant ECMO.

Abbreviations: CIT = cold ischemic time; ECMO = extracorporeal membrane oxygenation; eGFR = estimated glomerular filtration rate; ICU = intensive care unit; LOS = length of stay; SOFA = Sequential Organ Failure Assessment.

**Figure S1**. Adjusted association between cold ischemic time and vasoactive-inotropic score at 24 hours after ICU admission using a 4-knot spline model (n=129)

Analyses were adjusted for recipient age, donor age, female-donor-to-male-recipient mismatch, pre-transplant LVAD.

Abbreviations: ICU = intensive care unit; LVAD = left ventricular assist device; VIS = vasoactive-inotropic score.

**Figure S2**. Overall survival by cold ischemic time (≥200 vs. <200 minutes)
